# Analysis of Non-Amyloidogenic Mutations in APP Supports Loss of Function Hypothesis of Alzheimer’s Disease

**DOI:** 10.3390/ijms24032092

**Published:** 2023-01-20

**Authors:** Meewhi Kim, Ilya Bezprozvanny

**Affiliations:** 1 Department of Physiology, UT Southwestern Medical Center at Dallas, Dallas, TX 75390, USA; 2 Laboratory of Molecular Neurodegeneration, Peter the Great St. Petersburg State Polytechnical University, 195251 St. Petersburg, Russia

**Keywords:** gamma-secretase, APP, Alzheimer’s disease, modeling, presenilins

## Abstract

Proteolytic processing of amyloid precursor protein (APP) plays a critical role in pathogenesis of Azheimer’s disease (AD). Sequential cleavage of APP by β- and γ-secretases leads to generation of Aβ40 (non-amyloidogenic) and Aβ42 (amyloidogenic) peptides. Presenilin-1 (PS1) or presenilin-2 (PS2) act as catalytic subunits of γ-secretase. Multiple familial AD (FAD) mutations in APP, PS1, or PS2 affect APP proteolysis by γ-secretase and influence levels of generated Aβ40 and Aβ42 peptides. The predominant idea in the field is the “amyloid hypothesis” that states that the resulting increase in Aβ42:Aβ40 ratio leads to “toxic gain of function” due to the accumulation of toxic Aβ42 plaques and oligomers. An alternative hypothesis based on analysis of PS1 conditional knockout mice is that “loss of function” of γ-secretase plays an important role in AD pathogenesis. In the present paper, we propose a mechanistic hypothesis that may potentially reconcile these divergent ideas and observations. We propose that the presence of soluble Aβ peptides in endosomal lumen (and secreted to the extracellular space) is essential for synaptic and neuronal function. Based on structural modeling of Aβ peptides, we concluded that Aβ42 peptides and Aβ40 peptides containing non-amyloidogenic FAD mutations in APP have increased the energy of association with the membranes, resulting in reduced levels of soluble Aβ in endosomal compartments. Analysis of PS1-FAD mutations also revealed that all of these mutations lead to significant reduction in both total levels of Aβ produced and in the Aβ40/Aβ42 ratio, suggesting that the concentration of soluble Aβ in the endosomal compartments is reduced as a result of these mutations. We further reasoned that similar changes in Aβ production may also occur as a result of age-related accumulation of cholesterol and lipid oxidation products in postsynaptic spines. Our analysis more easily reconciled with the “loss of γ-secretase function” hypothesis than with the “toxic gain of Aβ42 function” idea. These results may also explain why inhibitors of β- and γ- secretase failed in clinical trials, as these compounds are also expected to significantly reduce soluble Aβ levels in the endosomal compartments.

## 1. Introduction

Alzheimer’s disease (AD) is a major health problem for developed nations that has so far resisted the development of effective therapies. The amyloid hypothesis of AD indicates that accumulation of amyloidogenic Aβ42 peptide is a major driving force responsible for AD [1,2,3,4]. Both amyloidogenic Aβ42 peptide and non-amyloidogenic Aβ40 peptide are generated as a result of sequential proteolytic cleavage of amyloid precursor protein (APP) by β- and γ-secretases [5,6]. The amyloid hypothesis postulates that an increased ratio of Aβ42:Aβ40 levels is a key pathogenic event in AD [1,2,3,4].

γ-secretase is a multiprotein membrane complex composed of nicastrin, presenilin enhancer 2 (Pen-2), anterior pharynx defective 1 (Aph1) and presenilin [7,8,9]. In addition to APP, γ-secretases cleave various type I transmembrane proteins, including Notch receptor [10]. Most likely because of these additional substrates, pharmacological targeting of γ-secretase so far failed to yield effective therapy for AD [11,12]. Multiple clinical trials of γ-secretase inhibitors failed partly due to side effects resulting from the inhibition of cleavage of Notch receptor and other γ-secretase substrates, such as for example trial of semagacestat (LY-450139) [13]. Thus, there is a significant effort by the industry to develop “Notch-sparing” γ-secretase inhibitors that selectively block generation of Aβ42, but do not affect generation of Aβ40 or Notch receptor cleavage.

Processing of APP by γ-secretase occurs in plasma membrane and in early and late endosomal compartments [14,15,16,17,18]. In the previous study, we performed structural modeling of APP complex with γ-secretase [19]. Based on structural analysis of known γ-secretase structures [20,21], we proposed that APP can form a complex with γ-secretase in two potential conformations—M1 and M2. By analyzing effects of PS1-FAD and APP-FAD mutations, we proposed that M2 conformation, but not M1 conformation, of γ secretase complex with APP leads to amyloidogenic (Aβ42-generating) processing of APP [19]. We continue analysis of PS1-FAD and APP-FAD mutations on APP processing by γ-secretase in the present study. In our analysis, we focused on the hypothesis that AD causing mutations in both APP and presenilins act by reducing levels of soluble Aβ peptides in the endosomal compartments. This idea is an extension of “presenilin loss of function” hypothesis of AD that was proposed previously [22,23,24] and may help to explain why inhibitors of β and γ secretase have not been successful in clinical trials.

## 2. Results

### 2.1. Membrane Association of Aβ–Effect of Peptide Length

**Figure 1:**
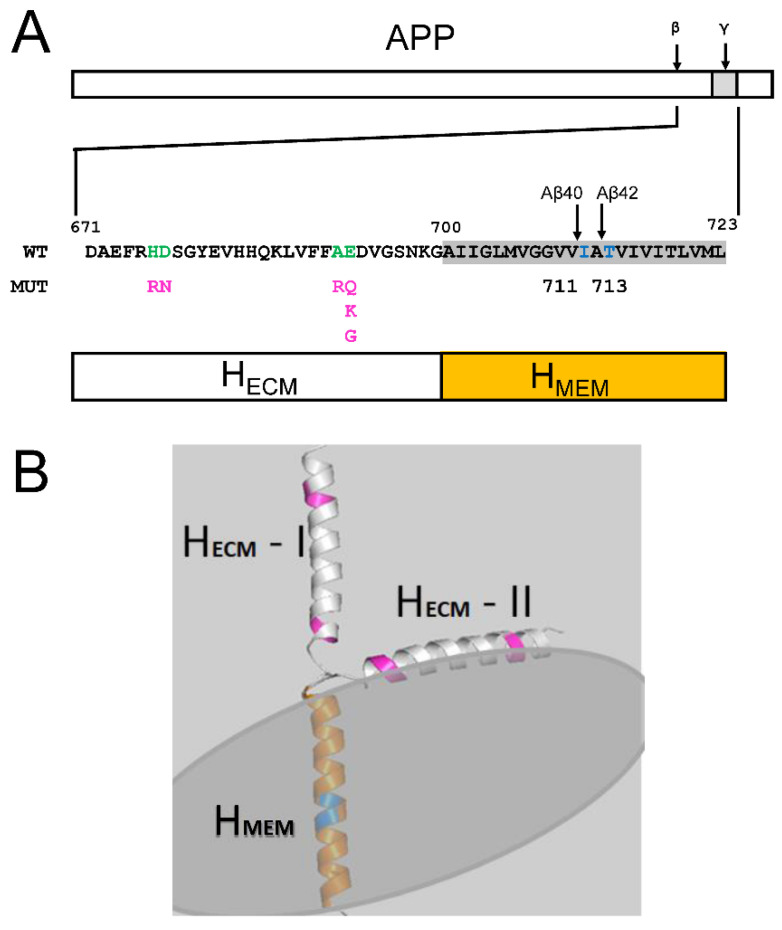
**Aβ peptide sequences and membrane-associated structures.** (**A**) Full-length APP protein is shown with locations of β- and γ-secretase cleavage sites as indicated. The wild type APP amino acid sequence is shown on the insert starting from β-secretase cleavage site (671) until the end of the transmembrane domain (723). Locations of γ-secretase cleavage sites resulting in generation of Aβ40 and Aβ42 peptides (711 and 713) are indicated by arrows. The bar diagram shows domain structure of Aβ peptide that consists of transmembrane (H_MEM_) and extramembrane (H_ECM_) α-helices. Locations and amino acid changes resulting from non-amyloidogenic APP-FAD mutations in H_ECM_ region are indicated below wild type sequence that is shown in green. (**B**) Models of Aβ peptide association with membrane in two different conformations of H_ECM_ α-helix. In model I H_ECM_ is perpendicular to the membrane, in model II H_ECM_ interacts with the membrane as a result of a 90 degree turn following H_MEM_. The positions of non-amyloidogenic APP-FAD mutations in H_ECM_ domain are indicated in pink. The orange-color region of H_MEM_ is a subject to the proteolysis by γ-secretase at positions indicated by blue for Aβ40 and Aβ42.

APP is initially processed by β-secretase at position 671 and then by γ-secretase within transmembrane domain at positions 711 (resulting in production of Aβ40) or 713 (resulting in production of Aβ42) (Figure 1A). Additional longer species of Aβ peptides can also be generated by γ-secretase cleavage sites beyond 713. The majority of APP processing occurs in endosomal membranes and, following generation of Aβ peptides, they partition between the membrane phase and intraluminal compartment of endosomes. Aβ partitioning is determined by its membrane-associating energy. In order to quantify the association of Aβ with the membrane, we calculated the membrane-association energy (*E_M_*) of generated peptides. The Aβ structural model used for *E_M_* calculation consists of two α-helical domains-extracellular matrix (ECM, H_ECM_) and membrane (H_MEM_) domains (Figure 1A). The H_MEM_ domain spans the membrane and the H_ECM_ adopts two conformations: membrane-associated (Model II) and membrane-dissociated (Model I) conformations (Figure 1B). The transmembrane region H_MEM_ is subjected to γ-secretase proteolysis (at sites shown by blue on Figure 1B).

**Figure 2:**
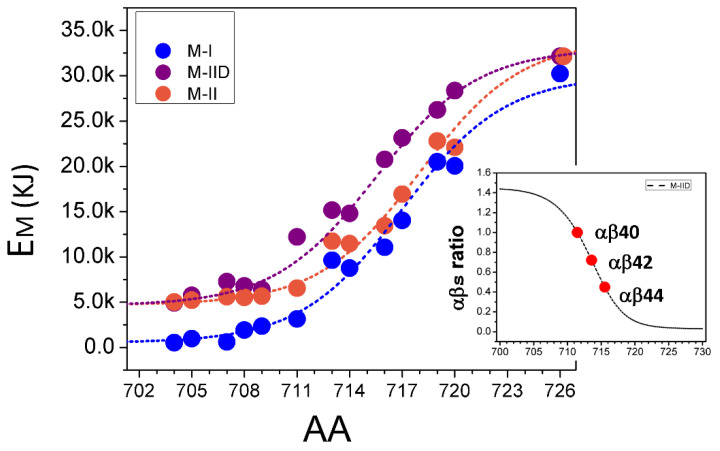
**The membrane-association energy of Aβ peptides.** A membrane-association energy (*E_M_*) of Aβ peptide is plotted as a function of Aβ length resulting from γ-secretase proteolysis between positions 704 and 725. *E_M_* is calculated for M-I (blue circles) and M-II (orange circles) conformations and for M-IID conformation that corresponds to M-II conformation with Asp residue added at carboxy-terminal end of Aβ peptide (purple circles). The results were fitted (smooth lines) using Equations (1) and (2). The insert shows predicted ratio of soluble and membrane associated Aβ peptides (αβs ratio) as a function of peptide size based on Equation (4). Red dots are the αβs values calculated for Aβ40, Aβ42 and Aβ44 as indicated.

The membrane-association energy (*E_M_*) for Aβ was calculated as a function of H_MEM_ peptides of different length (determined by position of γ-secretase cleavage site) and H_ECM_ for M-I and M-II conformations (Figure 2). The membrane-association energy *E_M_* consists of inner and surface interaction energies (*E_Inn_* and *E_Peri_*)

(1) $$E_{M}=E_{Peri}+E_{Inn}$$

The energy associated with transmembrane for Aβ peptide of size *Ab_i_*, *E_i,Inn_* is described with the Boltzmann equation below

(2) $$E_{i,\;Inn}=C_{Inn,}exp\lfloor \frac{Ab_{i}-Ab_{Inn}}{k}\rfloor$$

The constant values of *C_Inn_*, *Ab_Inn_* and *K* in Equation (2) are obtained by fitting the data on Figure 2, where results are plotted with blue circles for H_ECM_ in M-I conformation, orange circles for H_ECM_ in M-II conformation, and purple circles for H_ECM_ in M-II conformation with Asp modification at carboxy-terminal end. Asp modification at the carboxy-terminal end appears to cause a 3KJ shift in the energy of membrane association (Figure 2), suggesting that adding a charge to the carboxy-terminal of Aβ enhances Aβ partition from the membrane to the soluble phase. As expected, *E_M_* increases with an increase in the length of Aβ peptides due to the addition of hydrophobic residues from H_MEM_ (Figure 2). Interestingly, *E_M_* before residue I711 increases slowly (*E_M_*_,S_), and after I711 increases much faster (*E_M_*_,F_) (Figure 2). Thus, peptides longer than Aβ40 are expected to be significantly more membrane-associated. This difference is because *E_M_*_,S_ is determined by interaction of Aβ residues with the phosphate layer of the lipid bilayer, and *E_M_*_,F_ is determined by interaction of Aβ residues with acyl groups of the bilayer.

Comparison of Aβ membrane-association energy for M-I and M-II conformations of H_ECM_ indicates differences in their membrane-interaction properties. With the decreased size of H_MEM_, the *E_M_* approaches constant values for inner membrane association energy (*E_Inn_*). The value of *E_Inn_* is close to 0 KJ for H_ECM_ for M-I conformation and close to ~5 KJ for H_ECM_ for M-II conformation (Figure 2). These values determine peri-membrane-association energy (*E_Peri_*) of Aβ peptides. Another noticeable property of the results shown in Figure 2 is the transition point between *E_M_*_,S_ and *E_M_*_,F_ (T_E_) that occurs between residues I711 and A713. The *E_M_* at residue I711 for M-I and M-II conformations is similar to *E_Peri_*, while *E_M_* at residue A713 is increased by ~5 KJ (Figure 2). Thus, the membrane-association of Aβ makes a transition from primarily H_ECM_-dependent before residue I711 (Aβ40) to primarily H_MEM_-dependent after residue A713 (Aβ42). The increased *E_M_* with peptides starting with Aβ42 and longer suggests that most of these peptides remain membrane-associated (Ab_M_), reducing a soluble fraction of Aβ (Ab_S_).

Based on these considerations we built a mathematical model for the relationship between *E_M_* and the ratio between Ab_M_ and Ab_S_ for M-I and M-II conformations of H_ECM_. The *Ab_i_* in solvent (*Ab_i,s_*) relative to Aβ 40 is equal to

(3) $$Ab_{i,s}=Ab_{40,s}exp\lfloor \frac{-E_{M,i}+E_{M,40}}{E_{M,40}}\rfloor$$

(4) $$Ab_{i,s}/Ab_{40,s}=exp\lfloor \frac{-E_{M,i}+E_{M,40}}{E_{M,40}}\rfloor$$

The Equation (3) predicts that *E_M,i_* is inversely corelated with *Ab_i,S_*, so that larger *Ab_i_* has lower probability to be in solvent and higher probability to be membrane-associated. The relative value of *Ab_i,S_*/*Ab_40,s_* (Equation (4)) is shown as insert on Figure 2 as “αβs ratio” for *Ab_40,s_*, *Ab_42,s_* and *Ab_44,s_*.

### 2.2. Membrane Association of Aβ–Effect of Non-Amyloidogenic FAD Mutations in APP

**Figure 3:**
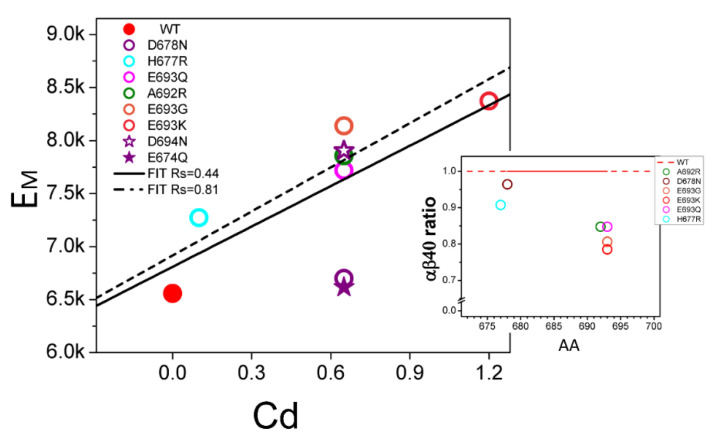
**Effect of non-amyloidogenic FAD mutations in APP located in H_ECM_ domain of Aβ.** The value of c_d_ for FAD APP mutations in H_ECM_ domain of Aβ is the difference in charge resulting from mutations and calculated at isoelectric point of wild type Aβ40. D694N and E674Q are artificial mutations used for control calculations. The linear fits yield regression coefficient Rs = 0.44 (sold line, all mutants data) and 0.81 (dashed line, with D678N replaced with D694N). The insert shows predicted changes in the ratio of soluble and membrane-associated Aβ40 peptides (αβs ratio) resulting from non-amyloidogenic FAD APP mutations in in H_ECM_ domain of Aβ.

From the analysis in the previous section, we concluded that Aβ42 and longer peptides associate with membranes significantly stronger than Aβ40 peptides. Based on this conclusion, we propose that reduction in levels of soluble Aβ contributes to AD pathology. To test this hypothesis, we analyzed the effects of non-amyloidogenic FAD mutations in APP located in the H_ECM_ domain of Aβ (Figure 1A) on membrane association of these peptides. Figure 3 shows sn *E_M_* of Aβ40 containing FAD APP mutations in the H_ECM_ domain. The effect of each mutation is represented as the resulting H_ECM_ charge difference (c_d_) determined at the isoelectric point of wild type Aβ40. The c_d_ values of all non-amyloidogenic FAD-APP mutations in the H_ECM_ domain shifted positively by +0.1~+1.2 when compared to the wild type Aβ40 sequence (c_d_ = 0) (Figure 3). The change in charge leads to increased *E_M_* (Figure 3), suggesting increased membrane association of Aβ40 containing FAD mutations in H_ECM_ domain. The correlation coefficient between FAD-induced charge difference c_d_ and membrane-association energy *E_M_* is equal to Rs = 0.44, a relatively weak correlation (solid line on Figure 3). The weak correlation is due to a single data point resulting from D678N mutation (Figure 3). To explain why D678N may be an outlier, we investigated a D678N-mutated H_CEM_ structure in Model II. We noticed that D678 residue is in the proximity of a positively charged R675 residue, which likely interacts directly with the negatively charged membrane. Therefore, negatively charged D678 residue is likely to be exposed to solvent, which explains why D678N mutation does not affect the membrane-association energy Aβ. To confirm the rational, we tested effects of D694N and E674Q mimic mutations (Figure 3, purple Stars). Importantly, D694 is surrounded by non-charged residues and E674 is neighbored with positively charged residues. The charge difference of both mimics is shifted positively by the same value as for D694N (purple stars on Figure 3). However, calculated *E_M_* for D694N is consistent with all other mutations, while E694N is consistent with D678N (Figure 3). Based on this analysis, we conclude that the uniquely small effect of D678N on *E_M_* is due to the neighboring charged residues. With this conclusion, we were able to recalculate the correlation between the charge difference and *E_M_* of Aβ40 by replacing D678N with D694N. After this correction, the correlation coefficient Rs is 0.81 (dotted line on Figure 3). The strong correlation indicates that FAD mutations in the resulting H_ECM_ domain of Aβ40 increase *E_M_* so that the mutant Aβ40 is expected to partition into the membrane more than wild type Aβ40 (Figure 3, insert).

In order to quantitatively describe the change in membrane association of FAD mutants of Aβ40, *E_M_* in Equation (1) was modified with *E_peri,,Mut_* for FAD-APP mutation.

(5) $$E_{Peri,MUT}=E_{Peri,WT}+a*c_{d,MUT}$$

Here, *c_d_* for the mutants is the charge difference at the isoelectric point when compared to the wild type Aβ40 sequence, and α is a linear regression coefficient from Figure 3:

(6) $$E_{M,mut}=E_{Peri,WT}+a*c_{d,Mut}+E_{Inn}exp\lfloor \frac{Ab_{i}-Ab_{Inn}}{k}\rfloor$$

The change of *E_M,Mut_* from *E_M_,_WT_* is

(7) $$E_{M,Mut}-E_{M,WT}=a*c_{d,mut}$$

Equation (7) describes the changes in *E_M_* of FAD-mutated Aβ40 as a result of charge difference *c_d_* induced by FAD mutations.

To predict the effects of FAD-APP mutation on the amount of Aβ in solvent (*Ab_S,Mut_*) the ratio of *Ab_S,Mut_*to *Ab_S,WT_* is derived from Equations (3) and (7).

(8) $$Ab_{s,\;Mut}=Ab_{s,\;WT}exp\lfloor \frac{-a*c_{d,Mut}}{E_{M,\;WT}}\rfloor$$

The relative *Ab_S,Mut_* to *Ab_S,WT_* for Aβ40 was calculated for each mutant using Equation (8) and is shown in Figure 3, Insert. *Ab_S,Mut_* (color dots) are all reduced when compared to *Ab_S,WT_* (Figure 3, Insert). The magnitude of FAD APP mutation effects on *Ab_S_* reduction for Aβ40 is comparable to the effects of Aβ42 (Figure 2, Insert). Thus, we concluded that the increase in the Aβ peptide length or non-amyloidogenic FAD mutations in the H_ECM_ domain enhance the membrane-association of Aβ and reduce the amount of Aβ in the endosomal soluble compartment to a similar degree.

### 2.3. Reduction in Soluble Aβ as a Result of FAD Mutations in Presenilin 1

**Figure 4:**
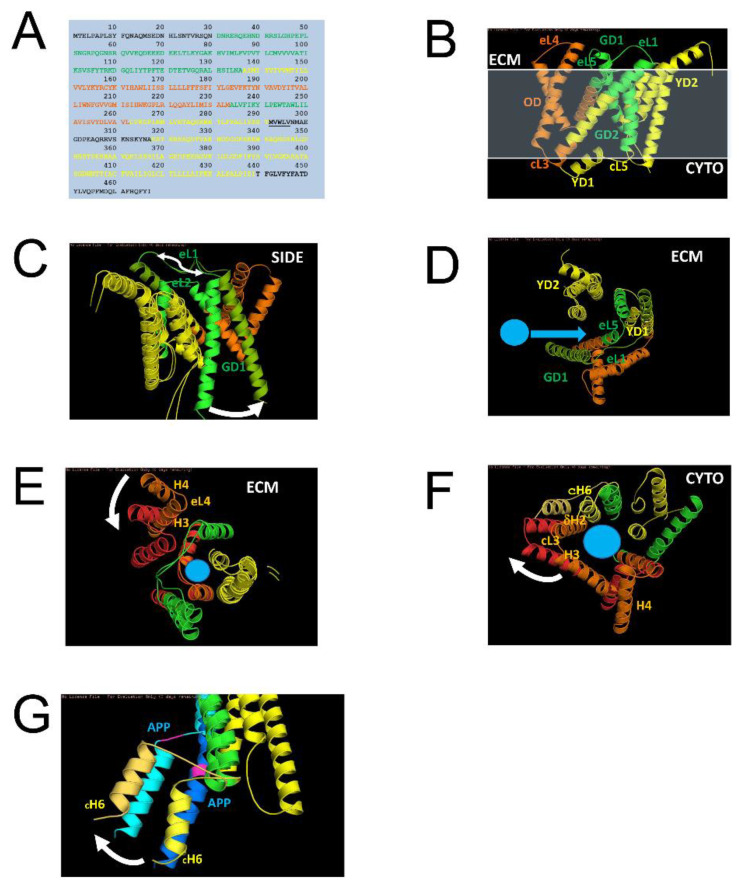
**FAD-PS1 and motions of PS1 during APP proteolysis.** (**A**,**B**) PS1 sequence (**A**) and structure (**B**) are color-coded for FAD-PS1 mutation groups defined in Table 1. GD is green domain, YD is yellow domain, and OD is orange domain. (**C**,**D**) The predicted motion (white arrows) of the green domain during APP cleavage—side view (**C**) and ECM view (**D**). The APP entry into active site of γ-secretase is shown by blue arrow on panel. (**D**–**F**) The predicted motion (white arrows) of orange domain during APP cleavage—ECM view (**E**) and cytosolic view. (**F**,**G**) The predicted motion (white arrows) of the yellow domain (_C_H6-YD) and APP (Blue) during APP cleavage is shown (side view). The cleavage site at VI residues of APP is shown in pink.

Aβ produced by APP proteolysis by γ-secretase. In the previous study, we used structural information to model a complex of γ-secretase with APP [19]. The proteolysis of APP is a dynamic process that involves changes in PS1 conformation [19]. To identify the local motions of PS1 involved in specific functions of the γ-secretase action on APP, we investigated the effects of FAD mutations in PS1 that affect the production of Aβ. Based on the analysis of the published data [25,26], we have been able to identify three groups of the FAD mutations (“green”, “orange” and “yellow”) located in the different domains of PS1 (Table 1, Figure 4). The green domains (GD) consist of green domain 1 (GD1) and green domain 2 (GD2). GD1 includes Helix1 (H1), part of Helix 2 (dH2) and their linker Loop1(_E_L1) in an extra cellular matrix (ECM) (Figure 4A–C). GD2 is composed of a transmembrane region that includes Helix 6 (H6), part of Helix 5 (dH5) and their linker Loop5 in ECM (_E_L5) (Figure 4A–C). The green domains are most dynamic in PS1 according to structural studies [21,27,28]. Yellow domains (YD) consist of YD1 that includes three most stable transmembrane helixes 7–9 (H7– H9), and YD2, which includes cytosolic part of H6 (cH6) and the interacting partner from the membrane region of Helix 2 (dH2) (Figure 4A–C). Amino-terminal YD1 and carboxy-terminal YD2 are assembled separately and stabilized by intradomain interactions. YD1 and YD2 together form a substrate binding site for APP (Figure 4D). cH6 of YD1 is linked to GD2 with _C_L6. The orange domain (OD) contains Helixes 3–4 (H3–H4) and a cytosolic part of H5 (cH5) (Figure 4A–C). The orange helixes are linked by two loops, a long Loop in ECM (_E_L3) and a short Loop in Cytosol (cL2).

Our analysis (Table 1) revealed that mutations in each of these groups have similar effects on the production and ratio of Aβ40/Aβ42, suggesting that the domains in each of these groups exert similar actions during APP proteolysis. The mutations in the “green” group lead to the greatest changes in the amount of total Aβ production, the mutations in the “orange” group result in the greatest change in the Aβ40/Aβ42 ratio, and the mutations in the “yellow” group have smaller effects on both total Aβ production and the Aβ40/Aβ42 ratio (Table 1). Importantly, all of these mutations lead to reduction in total amount of Aβ produced (Green~0.12, Orange ~0.42, and Yellow~0.51 when compared to the wild type PS1) and to reduction in Aβ40/Aβ42 ratio (Green~3.8, Orange ~2.7, and Yellow~4.5 when compared to the wild type PS1 value of ~10.0) (Table 1). In particular, “green” mutations nearly lost the activity of γ-secretase (~12% of WT), in agreement with the “loss of γ-secretase function” hypothesis [23]. Table 1 also shows that wild type PS1 generates about 10 time more Aβ40 than Aβ42, and that all PS1-FAD mutants reduce the Aβ40/Aβ42 ratio to less than 50% of the wild type (Green~3.8, Orange~2.7, and Yellow~4.5). The reduced production of Aβ peptides and reduced Aβ40/Aβ42 ratio should lead to a dramatic reduction in the levels of soluble endosomal Aβ, in agreement with our overall hypothesis.

### 2.4. Effects of Membrane Curvature on APP Processing by γ-Secretase

In the previous study, we proposed that the dynamic movements of the PS1/APP complex is facilitated by membrane shape and that membrane curvature has important effects on γ-secretase activity [19]. As Aβ is produced by the dynamic motions of γ-secretase, we modeled dynamic motions of each individual domain in each color group, as they are likely to be related to specific functions of γ-secretase. We determined that the configuration of GD1/2 linked by long _E_L1/5 is adjustable for membrane compression/expansion mostly at the extracellular matrix (ECM). This motion of GD1/2 enabled by _E_L1/5 allows a freely moveable arrangement of H1 with _E_L1, which opens a large gate and allows for APP to access the binding site from the membrane (Figure 4C,D). This motion of GD with _E_L1 for sensing the membrane at ECM and opening the gate is linked to the reduced Aβ production as a result of FAD mutations in GD domain of PS1 (Table 1). The loss of function linked with mutations in GD domain suggests that the sensing motion is involved in recruiting APP substrate (Figure 4D), and the dysfunction in recruiting APP leads to the reduced Aβ production. Therefore, the local motion of GD can be assigned to recruit motion sensing (RMS) of PS1 in the plasma membrane at ECM.

The “orange domains” (OD1/2) neighboring with RMS-GD are linked with loops (_C_L2/_E_L3) at cytosol and ECM (Figure 4E,F), which allow us to sense the membrane at ECM and cytosol. The _C_L2/_E_L3 could adequately sense the membrane at opposite side. In addition, dH2 interacts with _C_H6 of APP-binding YD1, whose motion was predicted to have a direct effect on Aβ40 generation by early endosome in the previous studies [14,15,16,17,18,19]. The motion of OD could be simulated with PS1 structure in the endosomes with double layers compressed at ECM and expanded at cytosol. The plausible movement of OD in the endosome is shown in Orange and Red models with directions in white arrow (Figure 4G). The protruding H4 and H3 (Orange OD in Figure 4E, ECM View) at sensing the compression by _E_L3 could be toggled onto the rest of PS1 domain (RED OD in Figure 4F, ECM View) in response. The ECM motion of H4/_E_L3/H3 (RED OD in Figure 4E, ECM View) could be synchronized with the motion of H2/_C_L2/H3 domain (Orange in Figure 4F, cytosolic View), which stretches along the expanding membrane layer at cytosol (RED in Figure 4F, cytosolic View). The white arrows in Figure 4G show that the protein interaction of dH2-OD1 with _C_H6 is to transfer the motion of OD to APP binding to _C_H6 of YD1. This motion conveyed to APP via _C_H6 is linked to the greatest change in the ratio of Aβ40/Aβ42 by FAD-PS1 mutants in OD (Table 1). This suggests that the local motion of OD is directly associated with the proteolysis motion by sensing membrane (PMS) at cytosol and ECM for Aβ generation. The APP binding site (ABS), YD1/2 are constructed with stable protein interactions, which are more sensible to the motions of OD and GD and less to the membrane. This explains the effects on Aβ production by FAD-PS1 mutations of YD (Table 1), which has the lowest effects on the Aβ production and the Aβ42:Aβ40 ratio when compared to FAD-PS mutation in OD and GD domains.

## 3. Discussion

### Loss of Aβ40 Function and AD

Previous studies of conditional PS1 knockout mice phenotype laid the foundation to the “loss of γ-secretase function” hypothesis of AD [22,23,24]. However, this hypothesis has rarely received acceptance in contrast to the “amyloid hypothesis” that assigns gain of toxic function to Aβ42 peptides, amyloid plaques and Aβ oligomers [1,2,3,4]. Interestingly, inhibitors of β- and γ- secretase were effective in inhibiting production of Aβ42, but have not been able to rescue cognitive performance in the AD patients treatment groups [29]. Actually, in many clinical trials of β- and γ-secretase inhibitors, cognitive performance of the treatment group was inferior to the placebo group. These clinical observations appear to contradict “amyloid hypothesis” and indirectly support “loss of γ-secretase function” hypothesis of AD.

In this paper, we would like to propose a hypothesis that may potentially reconcile these divergent observations. Specifically, we propose that the presence of soluble Aβ peptides in endosomal lumen (and secreted to the extracellular space) is essential for synaptic and neuronal function. Our analysis suggests that Aβ42 and longer Aβ species partition to the membrane much more easily than Aβ40 (Figure 2), and that reduction in the Aβ40/Aβ42 ratio leads to effective reduction in soluble Aβ in the endosomal compartments even when the same level of total Aβ is produced. Indirect support from this hypothesis can be obtained from the analysis of non-amyloidogenic FAD mutations in APP, all of them enhancing the association of Aβ40 with the membrane (Figure 3) and reducing its concentration in the endosomal lumen. Interestingly, analysis of PS1-FAD mutations also revealed that all of these mutations lead to significant reduction in both total levels of Aβ produced and in the Aβ40/Aβ42 ratio (Table 1), suggesting that the concentration of soluble Aβ in the endosomal compartments will be reduced as a result of these mutations. This is particularly true for the mutations in the “green” group, which on average display an almost 10-fold reduction in the levels of total produced Aβ (Table 1). Such data are more easily reconciled with the “loss of γ-secretase function” hypothesis than with the “toxic gain of Aβ42 function” idea. These results may also explain why inhibitors of β- and γ- secretase failed in clinical trials, as these compounds also expected to significantly reduce soluble Aβ levels in the endosomal compartments.

**Figure 5:**
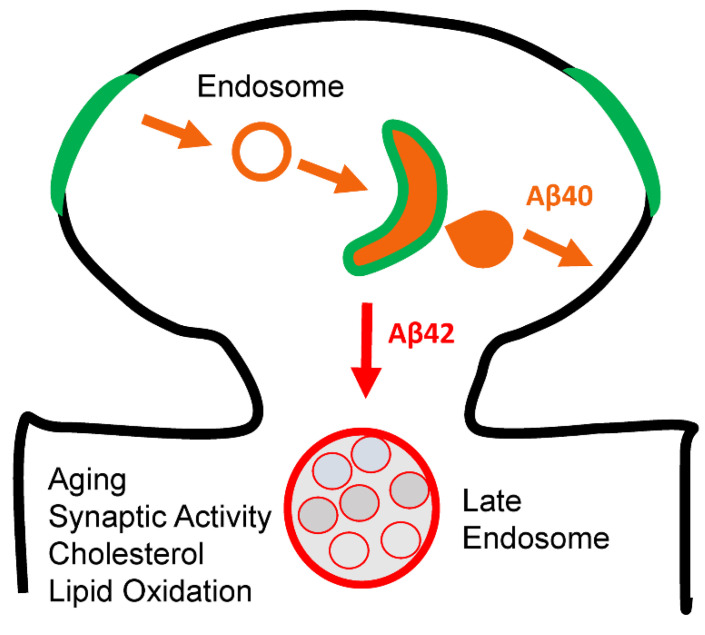
**Model of Aβ biogenesis in the postsynaptic spines.** Green membranes support active GD motion, orange membranes support active OD motion, orange areas favor accumulation of soluble Aβ40 and red membranes favor accumulation of Aβ42.

Proposed hypothesis may also be relevant not only for FAD but also for sporadic disease. Aging is a major risk factor for sporadic AD and we propose that aging-related factors such as accumulation of cholesterol and lipid oxidation may also induce reduction in the levels of endosomal soluble Aβ in postsynaptic compartments (Figure 5). These age-related changes in the membrane composition can affect γ-secretase function so that production of Aβ is impaired. Our analysis of conformational changes of PS1 (Figure 4) suggested that strongly curved membranes such as in early or late endosomes favor production of Aβ, but flat and rigid membranes such as plasma membrane do not. It is therefore likely that age-related accumulation of cholesterol and lipid oxidation products may lead to cellular membranes becoming more rigid and flat, reducing activity of γ-secretase and production of Aβ40 (Figure 5).

Although at the moment we are not certain what essential function is played by soluble Aβ in the brain, some recent experimental evidence is consistent with this hypothesis [30]. In experiments with iPS-derived human neuronal cells, these investigators concluded that Aβ at physiological concentrations supports synapse function in human neurons. Our analysis indirectly supports this hypothesis and suggested that familial and sporadic AD may be related to loss of this putative function of soluble Aβ due to reduced activity of γ-secretase and increased partitioning of Aβ species to the endosomal membrane compartment. The main significance of our findings is that our analysis may help to reconcile the “loss of γ-secretase function” hypothesis with the “toxic gain of Aβ42 function” idea. Our results may also explain why inhibitors of β- and γ- secretase failed in clinical trials, as these compounds are also expected to significantly reduce soluble Aβ levels in the endosomal compartments.

## 4. Materials and Methods

### 4.1. The Aβ Peptide Model Building and Membrane-Associating Energy (*E_M_*) Calculations

The structure of the Aβ peptide was built in PDB format from two α-helical domains, H_ECM_ and H_MEM_, using Coot program v0.9.8.1 [31]. The α-helix in ECM (H_ECM_) was built in two conformations, one bound to the membrane surface (Model II) and the other is in free form (Model I). The different peptide sizes of Aβ in both conformations were generated based on published reports [26,32,33,34]. The FAD-APP mutations were introduced to Aβ40 sequence in M-II conformation using Coot. Resulting PDB files were used to calculate EM by MODA with adding electrostatics [35]. Carboxy-terminal Asp modification PDB model of Aβ40 was also generated in order to mimic the negative charge effect on *E_M_*. Changes in charges resulting from APP-FAD mutations were calculated as a function of pH using PROTEIN CALCULATOR v3.4. The charge difference due to FAD-APP mutations (Cd) was read at isoelectric point of wild type Aβ40.

### 4.2. Classification of PS1-FAD Based on Generated Aβ Products

The properties of generated Aβ products for each PS1-FAD mutant were obtained from the published reports [25,26]. The levels of Aβ40 and Aβ42 produced by each PS1-FAD mutant were normalized to the levels produced by wild type PS1. Based in these data, each mutant was manually assigned to different “color group”—green (37 mutants), orange (38 mutants) or yellow (59 mutants). Members of “green” group had greatest changes in total Aβ production, members of “orange” group had greatest changes in Aβ40/Aβ42 ratio, and members of “yellow” group had relatively small changes in total Aβ production or Aβ40/Aβ42 ratio. The structure of PS1 was color-coded based on positions of each colored group members in PS1 sequence.

## Figures and Tables

**Table 1:** **Aβ peptide generation by FAD-PS1 mutants.** Based on the published reports [25,26] we identified 3 groups of PS1-FAD mutations—“green”, “orange” and “yellow”. For each group the levels of Aβ40 and Aβ42 produced PS1-FAD mutants were normalized to the levels produced by wild type PS1. The normalized values were averaged within each group and shown as mean (S.E., n = 37, 38, and 59 as indicated for each group). The total levels of Aβ for each group and wild type were calculated by adding average Aβ40 and Aβ42 values. An average Aβ40/Aβ42 ratio for each group and wile type was calculated by dividing mean Aβ40 to mean Aβ42 values.

Aβ Product	PS1 MUTANT Groups			WT
	GREEN (n = 37)	ORANGE (n = 38)	YELLOW (n = 59)	
**Aβ40 (norm)**	0.09189 (0.189)	0.30026 (0.037)	0.41525 (0.490)	1.0
**Aβ42 (norm)**	0.024 (0.027)	0.11187 (0.169)	0.0903 (0.09)	0.1
**TOTAL Aβ**	0.126	0.412	0.513	1.1
**Aβ40/Aβ42**	3.8	2.7	4.5	10.0

## Data Availability

Not applicable.
